# Fenton Discoloration of Ultrasonicated Purple Cactus Pear Juice

**DOI:** 10.3390/molecules22081344

**Published:** 2017-08-15

**Authors:** Isidro Reyes-Hernández, Nelly del S. Cruz-Cansino, Ingrid Renata Santander-Martínez, Ernesto Alanís-García, Luis Delgado-Olivares, Esther Ramírez-Moreno, José A. Ariza-Ortega, Ariana Omaña-Covarrubias, Jesús Martín Torres-Valencia, José de Jesús Manríquez-Torres

**Affiliations:** 1 Centro de Investigación Interdisciplinario, Área Académica de Nutrición, Instituto de Ciencias de la Salud, Universidad Autónoma del Estado de Hidalgo, Circuito Actopan-Tilcuautla s/n, Ex hacienda La Concepción, San Agustín Tlaxiaca, Hidalgo 42160, Mexico; isidro_nutri_reyes@hotmail.com (I.R.-H.); ncruz@uaeh.edu.mx (N.d.S.C.-C.); renatasantander01@gmail.com (I.R.S.-M.); ernesto_alanisgarcia@hotmail.com (E.A.-G.); dolito2@hotmail.com (L.D.-O.); rme1234@yahoo.com (E.R.-M.); jose190375@hotmail.com (J.A.A.-O.); annaira76@hotmail.com (A.O.-C.); 2 Área Académica de Química, Universidad Autónoma del Estado de Hidalgo, Km 4.5 Carretera Pachuca-Tulancingo, Mineral de la Reforma, Hidalgo 42184, Mexico; jmtv.np@gmail.com

**Keywords:** cactus pear, juice, ultrasound, color, betalains, Fenton reaction

## Abstract

The aim of this study was to evaluate the stability of color, betaxanthin, and betacyanin pigments in the presence of Cu(II)-dependent hydroxyl radicals (HO•) from ultrasonicated purple cactus pear juice at amplitudes of 40%, 60%, and 80%, in comparison to untreated sample. L* parameter of juice treated at 40% and 80% amplitude for 25 and 15 min, respectively (11.3 and 9.3, respectively), were significantly higher compared to the control; b* and hue parameters of juice treated at 80%, 25 min showed values of 1.7 and 0.1, respectively. Color differences (Δ*E*) were lower (<3) for juices treated at high amplitude (80%) and short times (3–5 min). Juice treated at 40% 15 min, 60% 25 min, 80% 15 and 25 min presented high values of betacyanins (281.7 mg·L^−1^, 255.9 mg·L^−1^, 294.4 mg·L^−1^, and 276.7 mg·L^−1^, respectively). Betaxanthin values were higher in the juices treated at 40% 5 min and 80% 15 and 25 min (154.2 mg·L^−1^, 135.2 mg·L^−1^, and 128.5 mg·L^−1^, respectively). Purple cactus pear juice exhibited significant chelating activity of copper ions and great stability when exposed to HO•.

## 1. Introduction

Ultrasound is an alternative technology to conventional thermal treatment and is characterized by the generation of longitudinal waves when a sonic wave meets a liquid medium, creating regions of alternating compression and explosion [1]. When high-power ultrasound propagates in the liquid, changes in pressure generate cavitation bubbles that collapse violently in the succeeding compression cycles of a propagated sonic wave. Several mechanisms act when ultrasound is applied to fluids; i.e., bubble implosions that lead to thermal effects or microstreaming, and implosion shock waves that produce mechanical stresses [2] promoting the release of cellular compounds and increase of liquid temperature [3]. Ultrasound technology applied to cactus pear juice has a minimal effect on nutritional quality parameters such as contents of phenols and ascorbic acid or antioxidant activity; moreover, ultrasound may even increase the release of some of these compounds [3,4].

Cactus pear (*Opuntia ficus indica*) in Mexico can be cultivated in semi-arid areas that offer limited growth possibilities for other fruits and vegetables, but it accounts for more than 45% of the worldwide production [5]. Previous studies reported that ultrasound in purple and green cactus pear juices significantly reduces microbial load without affecting its quality and antioxidant parameters [3,4,5,6]. On the other hand, this fruit is rich in phytochemicals, such as betalains which have important biological activity and are rarely found in other edible vegetables; betalains are found in roots, fruits and flowers. The few well-known edible sources of betalains are red and yellow beets (*Beta vulgaris L. ssp. vulgaris)*, Swiss chard (*Beta vulgaris L. ssp. cicla*), leaf amaranth or cereal (*Amaranthus sp.*), and cactus fruits from the genus *Opuntia* and *Hylocereus* [1,2,3,4,5,6,7]. Betalain pigments are also known as safe food colorants [8,9]. In addition, the phenolic and amine groups confer these pigments with reducing and radical-stabilizing properties that make them potent antioxidants. Betalains are advantageous over anthocyanins because of their superior stability as natural colorants at pH 4–7, as well as their potential to prevent degenerative diseases such as cancer, diabetes, and cardiovascular diseases [10,11]. A nutritional study revealed that supplementation with cactus pear fruits for at least two weeks decreased the level of plasma markers of oxidative stress and lipid hydroperoxides of circulating low-density lipoprotein in healthy humans [12]. In addition, betalains also have beneficial effects on the redox-regulated pathways involved in cell growth and inflammation [13,14].

Color plays a key role in consumer acceptance of food [15], but is a parameter sensitive to thermal processing; thus, colorants may be used to restore the initial appearance or accentuate food color to meet consumer expectations [16]. The most abundant pigments in cactus pear are the red-purple betanins (a type of betacyanins) [17,18]. Oxidation causes aging, physical damage, viral infection, and prompts the liberation of toxic substances such as free radicals (O_2_•^−^, H•, and HO•) [19]. The latter (HO•) is prevalent in in vivo aqueous environments and easily crosses cell membranes at specific sites [20]. The classic mechanism of the Fenton redox reaction involves the oxidation of Fe^2+^ ions to Fe^3+^ and the reduction of H_2_O_2_ to a hydroxyl radical and hydroxide ion. In a similar way, Cu also acts as a catalyst in the decomposition of H_2_O_2_ (Cupro-Fenton reaction) [21,22]. Both transition metals react with H_2_O_2_ to form an intermediate complex that then decomposes, forming the radical HO• [21]. Betacyanins in cactus pears show great stability when exposed to HO• [23,24,25], but it is unknown how ultrasound treatment may affect this stability in a liquid matrix (cactus pear juice).

Therefore, the purpose of this study was to evaluate the color, browning index, betalains content, and stability of betacyanins in the presence of Cu(II)-dependent hydroxyl radicals (HO•) in purple cactus pear juice after different ultrasound treatments.

## 2. Results and Discussion

### 2.1. Color and Browning Index

Color parameters L*, a*, b*, chroma, hue, Δ*E*, and browning index (A 420 nm) obtained at different ultrasound conditions are shown in Table 1. L* values for juice treated at 40% 25 min and 80% 15 min were significantly higher (11.3 ± 3.8, 9.3 ± 4.2, respectively) than the control, and most other samples which exhibited values between 4.5–8.0. Sonication time and high amplitude (%) affected the L* value of juice. According to Tiwari et al. [26], time affects lightness because color pigments in the juice are exposed for longer periods to high shear forces in the vicinity of collapsing bubbles. Our results showed that lightness was also affected by high amplitudes, although sonication times were shorter. The increased lightness observed for some treatments may also be attributed to the partial precipitation of unstable suspended particles [27].

Color parameter a* (red-green axis) was positive for all treatments, implying that cactus pear juice was within the red color. Ultrasound-treated juices presented similar values to the control, in the range of 21.1–25.9, and no significant differences were observed (*p* > 0.05). Parameter b* (yellow–blue axis) was also positive for all samples within the yellow color axis. Juice treated at 80% 25 min exhibited the highest b* value (*p* < 0.05) among all samples, whereas no differences were observed between the other treatments and the control. A similar slight increase of b* was reported by Tiwari et al. [26] for orange juice treated by ultrasound. Parameter C* indicates the degree of saturation, purity, or intensity of visual color [28]. In our study, results demonstrated that ultrasound did not affect the C* value of the treated juice, which ranged from 21.1 ± 3.4 to 28.4 ± 2.3. Hue (h°) represents tonality, and was significantly higher for treatment at 80% 25 min (0.06 ± 0.01) compared to all other samples that showed values between 0.01–0.03. Changes in parameters L*, a*, and b* agreed with the calculated total color difference (Δ*E*) for each treatment. Minimum and maxim Δ*E* observed among juices were between 2.5 and 6.4, with the samples treated at 80% amplitude for short times (3–5 min) exhibiting the lowest values (<3). Applied ultrasound amplitudes and times may explain these differences between experimental samples and the control; for instance, higher power ultrasound forms larger bubbles that collapse less violently, which may reduce cavitation effects [29]. Cavitation could be responsible for color changes observed among juices by means of various physical, chemical, or biological effects such as the acceleration of chemical reactions, increased diffusion rates, dispersion of aggregates, or breakdown of susceptible particles such as enzymes and micro-organisms [1].

Regarding browning index, significant differences between ultrasound and control samples were not observed, and values ranged between 0.5 and 0.8. Yuan et al. [30] reported that browning after ultrasound treatment is attributed to the sugar content; in previous studies on cactus pear juice, total solids and browning index remained unchanged after ultrasound treatment [3]. Non-enzymatic browning may result from the condensation of carbonyl groups with amino acids, or the reaction of sugars and ascorbic acid in the absence of free amino acids (caramelization) [2].

### 2.2. Betalains

**Figure 1:**
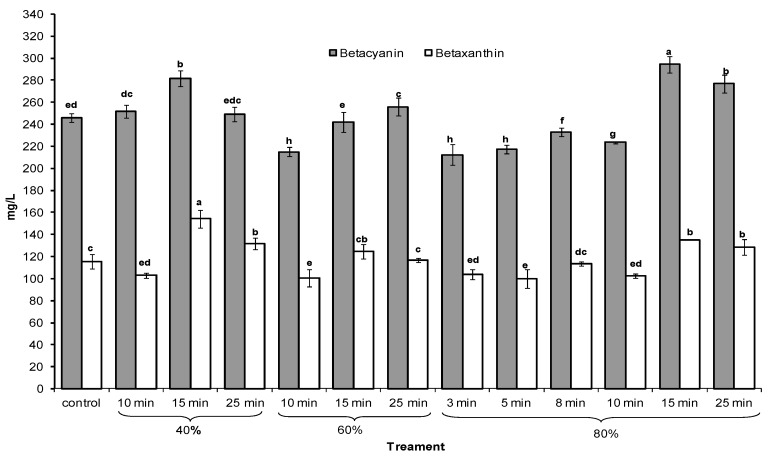
Betacyanins (mg betanin equivalent (BE)/L) and betaxanthins (mg indicaxanthin equivalent (IE)/L) content in purple cactus pear juices treated at different ultrasound conditions. a–h different letters in the same bar indicate significant differences (*p* < 0.05).

Betalains are natural pigments of chemotaxonomical significance which are typically associated with plants of the order caryophylales [31]. These water-soluble pigments are of great interest for their antioxidant activity, which has been evaluated in several studies [32,33,34]. Two betalain derivatives are present in cactus pears: betanin and betaxanthin, responsible for the red-purple and yellow-orange color, respectively, and have antioxidant activity without toxic effects in humans [24,35,36]. Betalain contents (betacyanins and betaxanthins) in purple cactus pear juice are shown in Figure 1. The control presented similar betacyanins values (245.9 ± 6.5 mg·L^−1^ expressed as betanin equivalent, BE) to those reported by Sumaya-Martinez et al. [23] in cactus pear fruits. Treatments at 40% 10 min, 40% 25 min, and 60% 15 min showed similar values to the control. Ultrasound conditions exhibited the highest values at 40% 15 min (281.7 ± 8.2 mg·L^−1^), 60% 25 min (255.9 ± 1.9 mg·L^−1^), and 80% 15 and 25 min (294.3 ± 0.5 mg·L^−1^ and 276.7 ± 7.3 mg·L^−1^, respectively), while shorter treatment times at the highest amplitude showed the lowest values. Betaxanthin values in the control were 115.3 ± 3.8 mg·L^−1^. Juices treated at 40% 15 min (154.2 ± 11.0 mg·L^−1^) and 80% 15 min and 25 min (135.2 ± 7.5 mg·L^−1^ and 128.5 ± 8.2 mg·L^−1^, respectively) had the higher values. The increase of these pigments in treatments at 80% 15 min and 25 min could be attributed to the increase of total soluble solids due to the sonication. Zafra-Rojas et al. [3] described an increase of total soluble solids caused by the reduction of particle size during ultrasound thanks to the collapse of cavitation bubbles formed on the surface, and the disruption of biological cell walls which also facilitates the release of pigments [3]. On the other hand, betalains content is affected by numerous external factors, particularly time–temperature conditions during food manufacture [3], as well as temperature, light, and oxygen exposure during storage [37]. Some studies report a superior stability of betacyanins as compared to betaxanthins at room temperature [38] and after heating [39,40,41,42], besides exhibiting stability in the presence of oxygen [40].

### 2.3. Release and Stability of Betacyanins in the Presence of Hydroxyl Radicals (HO•)

**Figure 2:**
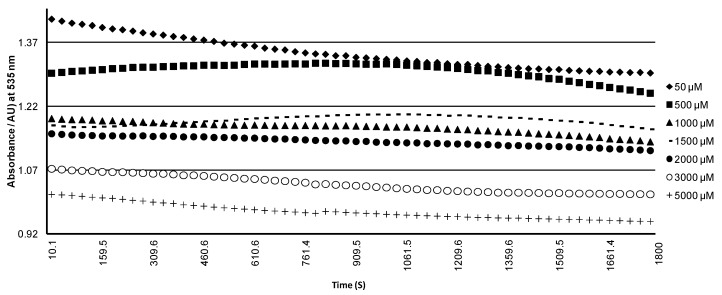
Betacyanins bleaching kinetics at different CuSO_4_ concentrations in purple cactus pear juices treated by ultrasound.

The Fenton reaction-based method detects non-enzymatic oxidation by assessing lipid peroxidation by oxygen free radicals [43]. The most important alternative theory of the Fenton reaction is based on the participation of free radicals as activated intermediates [44,45]. This explains the promotion effect by assuming the existence of the reaction between transition metals like copper Cu^2+^ and the free radical HO• [46]. According to the Fenton theory, during ultrasound treatment, radicals (HO• and •HO^2^ radical, Cu^2+^ ions) act as very reactive species and should be present in very low steady-state concentrations [46]. Ultrasound provokes several physical and chemical changes that lead to the formation of free radicals. However, our results showed that in extreme oxidizing conditions and the conditions used, purple cactus pear juice exhibited great stability when exposed to HO●. The effect of CuSO_4_ concentration on bleaching kinetics of purple cactus pear juice treated by ultrasound is shown in Figure 2.

Bleaching speed of betacyanin depends on the concentration of Cu^2+^ and increases with HO• radical concentration. A kinetic curve for the bleaching of betacyanin in the presence of HO● radical was obtained. Bleaching kinetics were adjusted to the exponential function, *y* = Ae − b*x*, which was fitted to the curve to calculate the speed of bleaching of betacyanin based on the concentration of CuSO_4_. The concentration of CuSO_4_ that can be added to the juice without significantly increasing the speed of bleaching would depend on the concentration of betacyanins and other compounds with antioxidant activities such as ascorbic acid and total phenolic compounds, since they protect betacyanins from oxidation [23].

**Figure 3:**
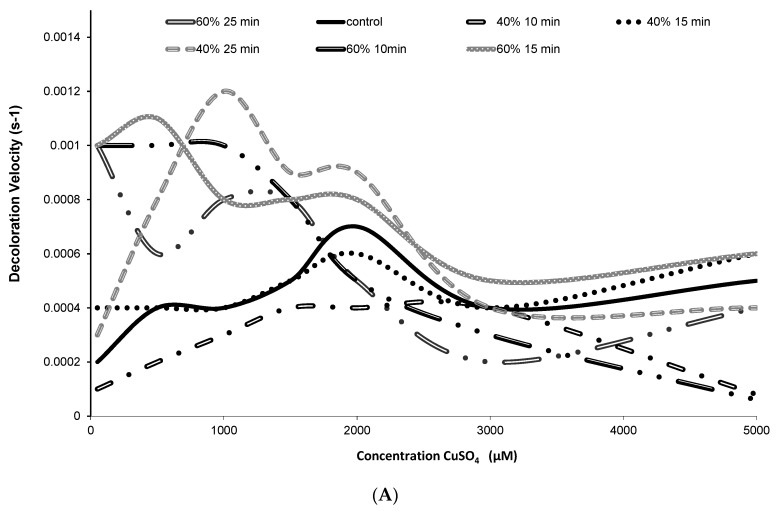

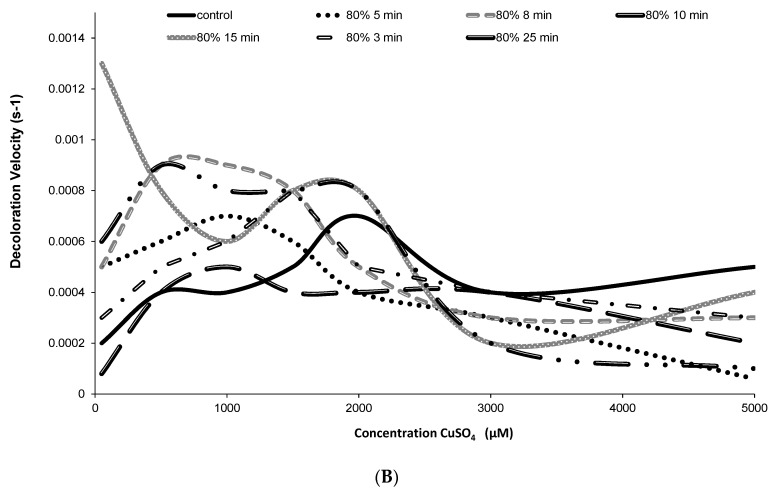
Bleaching speed of betacyanins in purple cactus pear juices treated by ultrasound at (**A**) amplitude of 40%, 60%, and 80% and (**B**) control sample at different times and CuSO_4_ concentrations.

Figure 3 shows the influence of CuSO_4_ concentration on the discoloration of the juice treated by ultrasound at different amplitudes and times. Concentrations up to 2000 μmol·L^−1^ CuSO_4_ induced a lower juice discoloration rate, particularly at 40% and 80% ultrasound amplitude at different times (Figure 3A,B).

The relationship between the extreme oxidation conditions used and the large amount of betacyanins released during ultrasound in cactus pear juice are similar to those reported by Sumaya-Martinez et al. [23]. Therefore, the increase in the release of these compounds resulted in a decrease in the rate of juice discoloration. The discoloration rate presents a fourth-order polynomial behavior between the maximum concentrations of CuSO_4_ (up to 2000 µmol·L^−1^) that can be added to the juice without significantly increasing the speed of bleaching of betacyanins due to the concentration of betacyanins in the samples. The treatments with lower discoloration rate were at concentrations lower than 2000 µmol·L^−1^ at an amplitude of 40% 10 min, 40% 15 min, and 80% 10 with R2 of 0.99, 0.92, and 0.98 respectively.

## 3. Materials and Methods

### 3.1. Preparation of Juice and Ultrasound Treatment

Purple cactus pears (*Opuntia ficus indica*) were obtained from a local market in Pachuca, Hidalgo, Mexico in spring 2016. Fruits free of external injuries were selected, washed, and manually peeled. To extract juice, the pulp was stirred using an industrial blender (model 38BL52 LBC10, Waring Commercial, Torrington, CT, USA) and then passed through a strainer to remove seeds. Juice samples were immediately treated by ultrasound (ultrasonic processor VCX-1500, Sonics & Materials, Inc., Newtown, CT, USA) at 1500 W with a constant frequency of 20 kHz, at 40%, 60%, and 80% amplitude levels for 10, 15, and 25 min with pulse durations of 2 s on and 4 s off. At the maximum amplitude (80%), treatment times of 3, 5, and 8 min were also included to evaluate their behavior within a short time; these conditions were based on previous study by Zafra-Rojas et al. [3]. Untreated juice was selected as control sample.

The intensity of ultrasound power—which dissipated from the probe tip—was calculated by following equation:

(1) $$I=\frac{P}{\pi r^{2}}$$

where *r* is the radius in meters of the probe tip and *P* is the input power level in watts. The input power was controlled through amplitude setting and the power level was adjusted to 40%, 60%, and 80% of total input power level (1500 J·s^−1^), which were equivalent to 600 J·s^−1^, 900 J·s^−1^, and 1200 J·s^−1^, respectively. The calculated sound intensities (expressed as sound energy rate per area units) were 1222.3 W·m^−2^, 1833.5 W·m^−2^, and 2444.6 W·m^−2^, respectively, and these corresponded to Pascals in sound pressure units.

### 3.2. Determination of Color

Color was measured using a Hunter Lab colorimeter (MiniScan XETM, Hunter associates Laboratory Inc., Reston, VA, USA) using the D65 illuminant with an angle of observation of 10°. Fifty milliliters of juice sample were tempered to 20 °C before analysis. Color was recorded using the CIE¾L*a*b* method, where L* indicates lightness (L* = 0 or 100 indicate black and white, respectively); chromaticity was measured as a* axis (−green to +red), and b* axis (−blue to +yellow). Numerical values of L*, a*, and b* were used to obtain Chroma (C = [a*2 + b*2]1/2) and hue angle (h°) (h° = tg^−1^ (b/a) [17]. Fresh juice samples were used as reference, and a higher Δ*E* represented greater color difference from the reference material [22], and was obtained from the following equation:

(2) $$\Delta E=\sqrt{\Delta L^{2}\;+\;\Delta a^{2}\;+\;\Delta b^{2}}$$

### 3.3. Browning Index (BI)

The browning index was measured in accordance with the method of Meydav et al. [15]. A 10 mL sample of juice was centrifuged (10 min, 756 g) at room temperature (Hamilton Bell Van Guard V650 BIOZARD, Montvale, NJ, USA). Five milliliters of ethyl alcohol (95%, Sigma-Aldrich, Dublin, Ireland) were added to 5 mL of juice supernatant and centrifugation was repeated under the same conditions. The absorbance of the supernatant was measured at 420 nm using a microplate reader (Power Wave XS UV-Biotek, software KC Junior, Winooski, VT, USA).

### 3.4. Determination of Betalain Content

Betacyanins and betaxanthin contents were determined according to Stintzing et al. [17] and Castellar et al. [25] and reported as milligrams of betanin equivalent (BE) per liter and milligrams of indicaxanthin equivalent (IE) per litre, respectively. Betacyanins and betaxanthins were quantified by reading absorbance at 535 nm and 484 nm, respectively, using a microplate reader (Power Wave XS UV-Biotek, software KC Junior, Winooski, VT, USA) and were calculated by the following equation:

(3) $$c=\frac{A\cdot DF\cdot MW}{\varepsilon \cdot l}\times 1000$$

where *c* is concentration of betacyanins or betaxanthins (expressed in milligrams per liter), *A* is absorbance at 535 nm or 480 nm, *DF* is dilution factor, *MW* is molecular weight, *ε* is extinction coefficient, *I* is width of the spectrophometer cell (0.316 cm). For betacyanin, *ε* = 60,000 L·mol^−1^ cm^−1^ and *MW* = 550 g·mol^−1^, and for betaxanthins *ε* = 48 L·mol^−1^·cm^−1^ and *MW* = 308 g·mol^−1^.

### 3.5. Stability of Betacyanin Pigments in the Presence of Hydroxyl Radicals (HO•)

A sample of 200 µL (control and ultrasound treatment), 88 µL of H_2_O_2_ (3%), and 1770 µL of 20 mmol·L^−1^ phosphate buffer at pH 6.8 were placed into 3 mL test tubes. Tubes were incubated at 30 °C in a temperature-controlled bath for 10 min in complete darkness. Then, 10 µL of CuSO_4_ were added at different concentrations (from 50 µmol·L^−1^ to 5000 µmol·L^−1^), and the absorbance was measured using a spectrophotometer at 535 nm every 30 s for a 30 min period. A kinetic curve for the bleaching of betacyanins in the presence of HO• radical was obtained. Bleaching kinetics were adjusted to the exponential function (function *y* = Ae − b*x*), where *y* is the absorbance at 535 nm, A is the value of absorbance at time 0, and *x* is the time in seconds, and were fit to the curve to calculate the speed of bleaching (b) based on the concentration of CuSO_4_.

### 3.6. Statistical Analysis

All values were obtained from three independent experiments, and each sample was analyzed in triplicate (*n* = 9) and expressed as means ± standard deviation (SD). The one-way analysis of variance (ANOVA) test was used to analyze the data, differences among means were compared by a Tukey test with a level of significance of *p* < 0.05, and stability of betacyanins was calculated based on the Pearson correlation matrix using the SPSS System for WINTM version 15.0.(Chicago, IL, USA)

## 4. Conclusions

In summary, minor differences were observed in most of the treatments in terms of L*, a*, b*, and hue values with respect to untreated juice. Some changes were observed in betacyanins (e.g., slight increase in juice treated at 80% 15 min). In general, ultrasound-treated cactus pear juice exhibited greater stability when exposed to HO•. This is because copper, reduced by hydrogen peroxide, can act as an electron donor, reducing the ferrous ions that are most active in the Fenton reaction. In addition, copper, when Cu^+^ is formed, can promote the formation of hydroxyl radicals (highly oxidizing species) in a Cupro-Fenton reaction.

## Figures and Tables

**Table 1:** Color parameters and browning index of purple cactus pear juice treated at different ultrasound conditions.

Treatment	L*	a*	b*	C*	h°	Δ*E*	Browning
control	4.5 ± 0.9 ^c^	21.1 ± 3.5 ^a^	0.7 ± 0.4 ^a^	21.1 ± 3.4 ^a^	0.03 ± 0.2 ^b^	–	0.5 ± 0.2 ^b^
40% 10 min	5.01 ± 0.62 ^c^	25.1 ± 2.3 ^a^	0.8 ± 0.2 ^a^	25.2 ± 2.3 ^a^	0.03 ± 0.0 ^b^	4.0 ± 1.4 ^abc^	0.7 ± 0.2 ^ab^
40% 15 min	6.3 ± 0.5 ^cb^	23.8 ± 3.0 ^a^	0.7 ± 0.6 ^a^	23.8 ± 3.0 ^a^	0.02 ± 0.0 ^b^	5.8 ± 2.2 ^ab^	0.7 ± 0.2 ^ab^
40% 25 min	11.3 ± 3.8 ^a^	21.8 ± 8.9 ^a^	0.4 ± 0.3 ^a^	21.8 ± 8.9 ^a^	0.02 ± 0.1 ^b^	4.1 ± 1.0 ^abc^	0.7 ± 0.3 ^ab^
60% 10 min	5.2 ± 0.6 ^c^	25.1 ±1.8 ^a^	0.8 ± 0.5 ^a^	25.1 ± 1.8 ^a^	0.03 ± 0.2 ^b^	4.2 ± 1.8 ^abc^	0.7 ± 0.1 ^ab^
60% 15 min	8.0 ± 4.1 ^cb^	25.6 ± 3.9 ^a^	0.4 ± 0.2 ^a^	25.6 ± 3.9 ^a^	0.01 ± 0.1 ^b^	6.3 ± 2.5 ^a^	0.5 ± 0.1 ^ab^
60% 25 min	6.6 ± 0.8 ^cb^	25.8 ± 2.8 ^a^	0.7 ± 0.2 ^a^	25.8 ± 2.8 ^a^	0.03 ± 0.0 ^b^	6.4 ± 1.7 ^a^	0.7 ± 0.1 ^ab^
80% 3 min	4.5 ± 0.7 ^c^	23.9 ± 2.9 ^a^	0.9 ± 0.2 ^a^	23.5 ± 3.3 ^a^	0.03 ± 0.0 ^b^	2.5 ± 1.0 ^c^	0.7 ± 0.1 ^ab^
80% 5 min	4.9 ± 0.7 ^c^	24.0 ± 3.2 ^a^	0.7 ± 0.1 ^a^	24.0 ± 3.2 ^a^	0.02 ± 0.0 ^b^	2.9 ± 1.0 ^cb^	0.8 ± 0.2 ^a^
80% 8 min	5.3 ± 0.7 ^c^	25.4 ± 2.3 ^a^	0.8 ± 0.4 ^a^	25.4 ± 2.3 ^a^	0.03 ± 0.0 ^b^	4.4 ± 1.1 ^abc^	0.6 ± 0.1 ^ab^
80% 10 min	5.5 ± 0.7 ^c^	25.9 ± 2.3 ^a^	0.6 ± 0.4 ^a^	25.9 ± 2.3 ^a^	0.02 ± 0.2 ^b^	5.5 ± 1.5 ^abc^	0.7 ± 0.1 ^ab^
80% 15 min	9.3 ± 4.2 ^ba^	21.9 ±4.3 ^a^	0.5 ± 0.3 ^a^	21.9 ± 4.3 ^a^	0.02 ± 0.1 ^b^	5.8 ± 2.4 ^ab^	0.7 ±0.7 ^ab^
80% 25 min	6.6 ± 0.5 ^cb^	28.3 ± 2.3 ^a^	1.7 ± 0.5 ^b^	28.4 ± 2.3 ^a^	0.06 ± 0.0 ^a^	5.8 ± 2.9 ^ab^	0.6 ± 0.3 ^ab^

^a, b, c^ different letters in the same line indicate significant differences (*p* < 0.05).
